# In Vitro Synergy of *Pongamia pinnata* Extract in Combination with Antibiotics for Inhibiting and Killing Methicillin-Resistant *Staphylococcus aureus*

**DOI:** 10.3390/antibiotics9030103

**Published:** 2020-02-29

**Authors:** Po-An Su, Shun-Lai Li, Hung-Jen Tang, Chi-Chung Chen, Ying-Chen Lu, Kuo-Chen Cheng, Yi-Chung Lin, Yin-Ching Chuang, Chih-Cheng Lai

**Affiliations:** 1 Department of Medicine, Chi Mei Medical Center, Tainan 71004, Taiwan; suboan0421@gmail.com (P.-A.S.); kcg.cheng@gmail.com (K.-C.C.); chuangkenneth@hotmail.com (Y.-C.C.); 2 Department of Pharmacy, Chia Nan University of Pharmacy & Science, Tainan 71710, Taiwan; 3 Department of Biotechnology, Southern Taiwan University of Science and Technology, Tainan 71005, Taiwan; shunlai@mail.stut.edu.tw (S.-L.L.); eg16958@yahoo.com.tw (Y.-C.L.); 4 Department of Medical Research, Chi Mei Medical Center, Tainan 71004, Taiwan; ccomm2@yahoo.com.tw; 5 Department of Food Science, National Chiayi University, Chiayi 60004, Taiwan; biolyc2016@gmail.com; 6 Departments of Medicine, Chi Mei Medical Center, Liou Ying, Tainan 73657, Taiwan; 7 Department of Internal Medicine, Kaohsiung Veterans General Hospital, Tainan Branch, Tainan 71051, Taiwan

**Keywords:** antibiotics, pharmaceuticals, resistance, staphylococcus, antimicrobial

## Abstract

Aims: Currently, we face the serious problem of multiple drug-resistant pathogens. The development of new antimicrobial agents is very costly and time-consuming. Therefore, the use of medicinal plants as a source of alternative antibiotics or for enhancing antibiotic effectiveness is important. Methods: The antibacterial effects of aqueous extracts of the seed coat of *Pongamia pinnata* (Linn.) Pierre in combination with several antibiotics against methicillin-resistant *Staphylococcus aureus* (MRSA) were tested by broth dilution, checkerboard, and time-kill methods. Results: For the combinations of *P. pinnata* with ampicillin, meropenem, cefazolin, cefotaxime, cefpirome, and cefuroxime, 70% to 100% were synergistic, with a fractional inhibitory concentration (FIC) index of < 0.5. For the time-kill method with 0.5× minimum inhibitory concentration (MIC) of *P. pinnata* in combination with 8, 4, 2, and 1 µg mL^−1^ of the various antibiotics, almost all of the combinations showed synergistic effects, even with the lowest concentrations of *P. pinnata*, except for aztreonam. No antagonistic effect was observed for these combinations. Conclusions: Based on these findings, aqueous seed coat extracts of *P. pinnata* have good potential for the design of new antimicrobial agents.

## 1. Introduction

The emergence of multidrug-resistant organisms (MDROs) has become a serious global health concern [1,2,3]. However, the development of new antibiotics to treat MDROs is both time-consuming and expensive. Therefore, the application of medicinal, plant-based, natural materials with antimicrobial activity provides another solution [4,5]. The ethnomedical plant *Pongamia pinnata* (Linn.) Pierre, which belongs to the family Leguminosae, is a glabrous, fast-growing tree in coastal and limestone areas of southern Taiwan and other tropical Asian countries [6,7]. Different parts of the *P. pinnata* tree have been widely used in making remedies for various types of infection [8,9,10] or inflammatory illnesses such as bronchitis and rheumatoid arthritis [11,12]. Based on the findings of previous studies [8,9,10,11,12], we studied the antimicrobial properties of *P. pinnata* against several gram-positive and gram-negative bacteria. In this preliminary investigation, we found that clinical isolates of pathogenic bacteria, including some MDROs, were susceptible to aqueous extracts of the seed coat of *P. pinnata* using the agar dilution method. Moreover, this aqueous extract exerted low minimum inhibitory concentrations (MICs) against *Staphylococcus* spp. (ranging from 0.39 to 0.78 mg mL^−1^). Therefore, this study was conducted to evaluate the synergistic effects of *P. pinnata* extract combined with other antibiotics against methicillin-resistant *Staphylococcus aureus* (MRSA). 

## 2. Materials and Methods

### 2.1. Bacterial Strains

Ten MRSA isolates from clinical specimens, including blood, joint fluid, and other aseptic specimens, were randomly obtained from the clinical microbiology laboratory of the Chi-Mei Medical Center. Staphylococcal species were identified based on colony morphology, Gram stain morphology, and coagulase test results and further confirmed by VITEK II methods. MRSA was stored at −70 °C in Protect Bacterial Preservers (Technical Service Consultants Limited, Heywood, Lancashire, England) before use.

### 2.2. Pulsed-field Gel Electrophoresis

**Figure 1:**
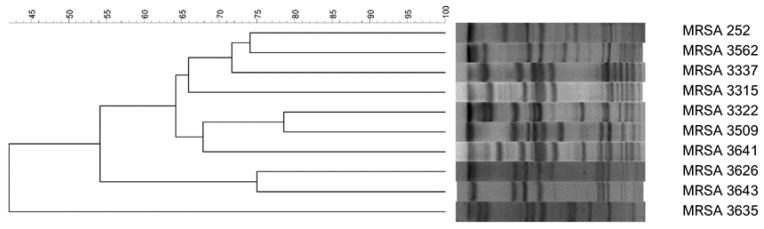
Pulsed-field gel electrophoresis patterns of all methicillin-resistant *Staphylococcus aureus* (MRSA) isolates.

PFGE was performed on *Staphylococcus aureus* DNA. In brief, bacterial chromosomal DNA was digested by using *SmaI* (New England Biolabs, Beverly, MA, USA). Electrophoresis was carried out for 22 h at 14 °C with pulse times ranging from 2 to 40 s at 6 V cm^−1^ using the Bio-Rad CHEF MAPPER apparatus (Bio-Rad Laboratories, Richmond, CA, USA). A dendrogram based on an unweighted pair grouping was generated by previously described methods [13]. Isolates that had > 80% similarity of their PFGE profiles were considered closely related strains according to the Dice correlation coefficient and the unweighted pair-group method, with averages within a position tolerance of 0.8% and an optimization parameter of 1% [14,15]. All ten MRSA isolates were confirmed as genetically unrelated based on the PFGE patterns (Figure 1).

### 2.3. Preparation of Aqueous Seed Coat Extracts

*P. pinnata* seeds were collected from dried fruits and surface-sterilized in 70% ethyl alcohol, and the seed coat was collected. Aqueous extracts were prepared by soaking the seed coat powder in 50 °C hot water for 24 hr and then evaporating it to dryness. 

### 2.4. Minimum Inhibitory Concentration (MIC) and Minimum Bactericidal Concentration (MBC) of P. pinnata Extract

The broth microdilution assay was used to determine the MIC and MBC. The crude extract was dissolved in type 1 ultrapure water at a concentration of 50 mg mL^−1^ and then diluted with Mueller-Hinton broth (Oxoid, Basingstoke, UK) to 6.25 mg mL^−1^. The inhibition of bacterial growth was defined as a clear well. The lowest concentration showing no turbidity change was considered the MIC. For determination of the MBC, 10 µL of liquid from each well was dropped onto Mueller-Hinton Agar and incubated at 37 °C for 24 h. The lowest concentration that showed no growth was taken as the MBC. Experiments were performed in triplicate and repeated twice.

### 2.5. Antibiotics

The antibiotics tested were ampicillin, cefazolin, cefuroxime, cefotaxime, and aztreonam (Sigma Chemical Co.), cefpirome (Hoechst), and meropenem (ICI Pharmaceuticals). MICs were determined by broth microdilution. Interpretation criteria for susceptibility testing were based on the Clinical and Laboratory Standards Institute guidelines [16,17,18]. The inoculum concentration was 5 × 10^5^ colony forming units (CFU) mL^−1^. The inoculated trays and plates were incubated in ambient air at 37 °C for 18–24 hr. The MIC was defined as the lowest antibiotic concentration that yielded no visible growth after overnight incubation. *S. aureus* American Type Culture Collection 29213 was included in each run as the standard quality control strain.

### 2.6. Time-kill Studies

MRSA 3322 was randomly selected. The time-kill method was performed according to the Clinical and Laboratory Standards Institute methodology [18]. In brief, bacterial suspensions were diluted to approximately 5 × 10^5^ CFU mL^−1^ in 25 mL of fresh Mueller-Hinton broth. In the time-kill study with *P. pinnata* only, 4-, 2-, 1-, and 0.5-fold MICs were tested, and bacterial numbers were determined at 0, 2, 4, 6, 8, 12, and 24 hours.

In the *P. pinnata* extract plus antibiotic combination time-kill studies, 0.39 mg mL^−1^ *P. pinnata* extract (0.5-fold MIC) was used as a reagent control. *P. pinnata* extract at 0.39 mg mL^−1^ plus 8, 4, 2, and 1 µg mL^−1^ of each antibiotic was tested to determine the combined effects.

Bacterial counts were measured at 0, 8, and 24 h by counting the colonies in 10-fold serially diluted 100 mL aliquots plated on nutrient agar (Difco Laboratories, Sparks, MD) and were incubated at 37 °C. Synergy was defined as a ≥ 2 log10 decrease in CFU mL^−1^ between the combination and its most active constituent after 24 h, with the number of surviving organisms in the presence of the combination ≥ 2 log10 CFU mL^−1^ less than the starting inoculum. Bacteriostatic and bactericidal activities were defined as 3 log10 and ≥ 3 log10 reductions in CFU mL^−1^ relative to that of the starting inoculum, respectively, at 24 h. All experiments were performed twice.

### 2.7. Checkerboard Microdilution Assay

The initial concentration of each bacterial suspension was 1.5 × 10^5^ CFU mL^−1^. The final drug concentrations ranges were 6.25–0.098 mg mL^−1^ for *P. pinnata*, 256–0.25 µg mL^−1^ for ampicillin, cefazolin, cefuroxime, cefotaxime, cefpirome, and aztreonam, and 32–0.25 µg mL^−1^ for meropenem. Two-fold dilutions of each drug or drug combination were tested. The results were read after plates were incubated at 37 °C for 24 hours. The following formulas were used to calculate the fractional inhibitory concentration (FIC) index: FIC of drug A = MIC of drug A in combination/MIC of drug A alone; FIC of drug B = MIC of drug B in combination/MIC of drug B alone; and FIC index = FIC of drug A + FIC of drug B. Synergy was defined as an FIC index of ≤ 0.5, indifference as an FIC index of > 0.5 but ≤ 4, and antagonism as an FIC index of > 4 [19].

## 3. Results

Table 1 provides the MIC and MBC values for the 10 MRSA isolates. The ratio of the MBC/MIC for the *P. pinnata* extract against the 10 MRSA isolates were all ≤ 4.

Table 2 shows the checkerboard microdilution results for each of the seven antimicrobial agents in combination with the *P. pinnata* seed coat extract against the 10 MRSA isolates. For the combinations of *P. pinnata* with ampicillin, meropenem and cefazolin, 10 out of 10 isolates were synergistic, with an FIC index ≤ 0.5. For the combination of *P. pinnata* with cefotaxime and cefpirome, 9 out of 10 isolates were synergistic; 7 out of 10 isolates were synergistic for the cefuroxime combination. Only the combination of *P. pinnata* extract with aztreonam had no synergistic effect, and the FIC index was 0.5 to 4. None of the combinations showed antagonism.

**Figure 2:**
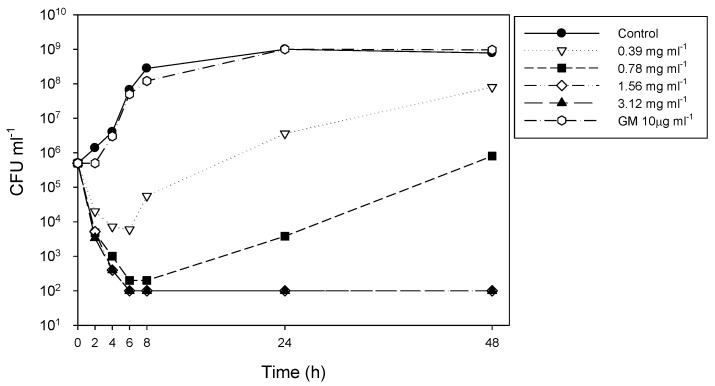
Time-kill results for the crude extract of *Pongamia pinnata* seed coat alone against methicillin-resistant *Staphylococcus aureus* 3322 incubated for 48 hr. The concentrations used were 0.5×, 1×, 2×, and 4× minimum inhibitory concentration, with an inoculum concentration of 5 × 10^5^ colony forming units (CFU) mL^−1^. Note. GM, gentamicin.

The suspensions of randomly selected isolates of MRSA 3322 were cocultivated with 0.5, 1, 2, and 4× the concentration of the respective MICs of the aqueous extracts for the time-kill assay. The inhibition effect persisted for 48 hours under the effect of 2× and 4× MIC (Figure 2).

**Figure 3:**
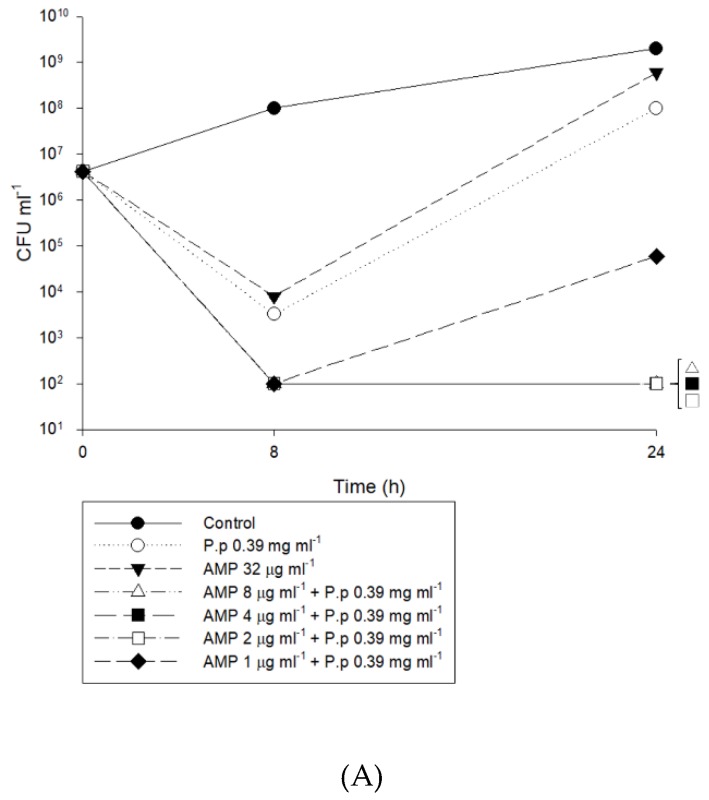

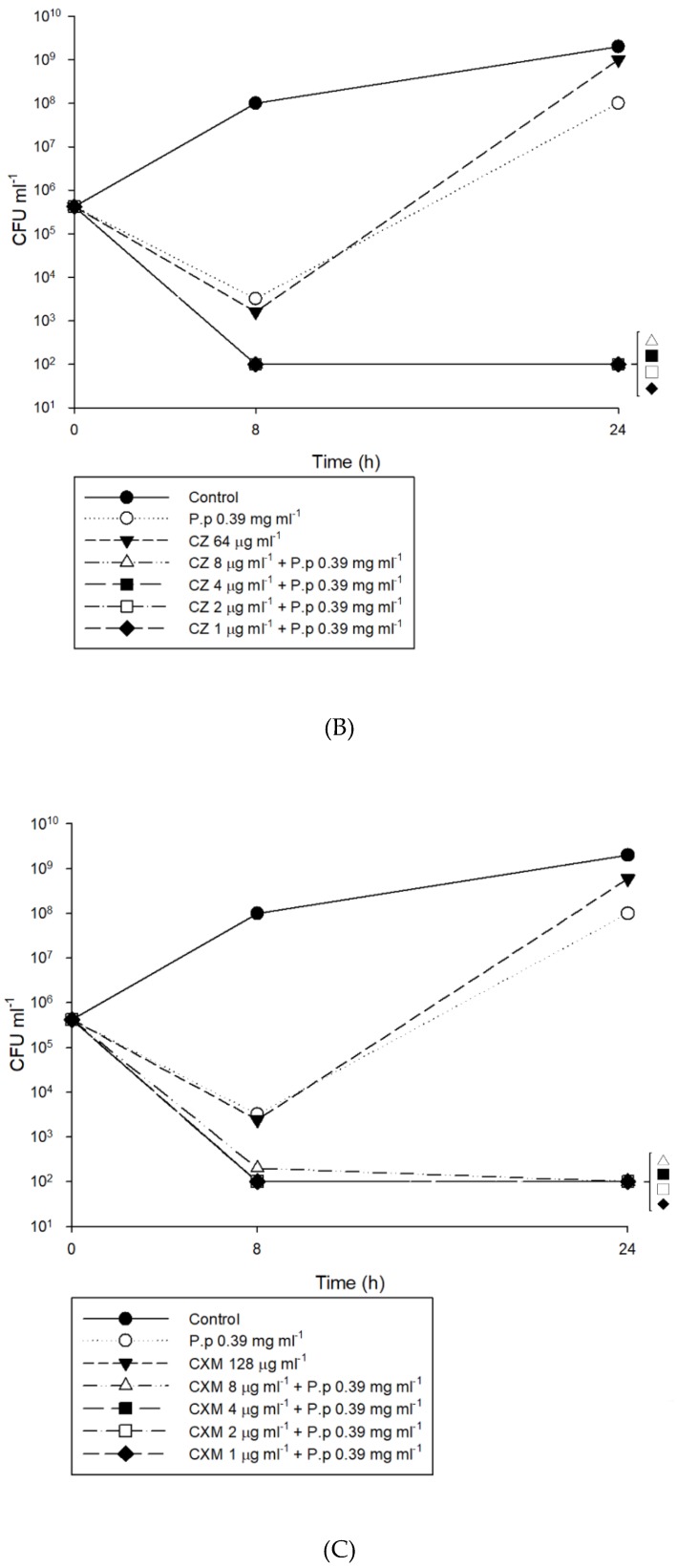

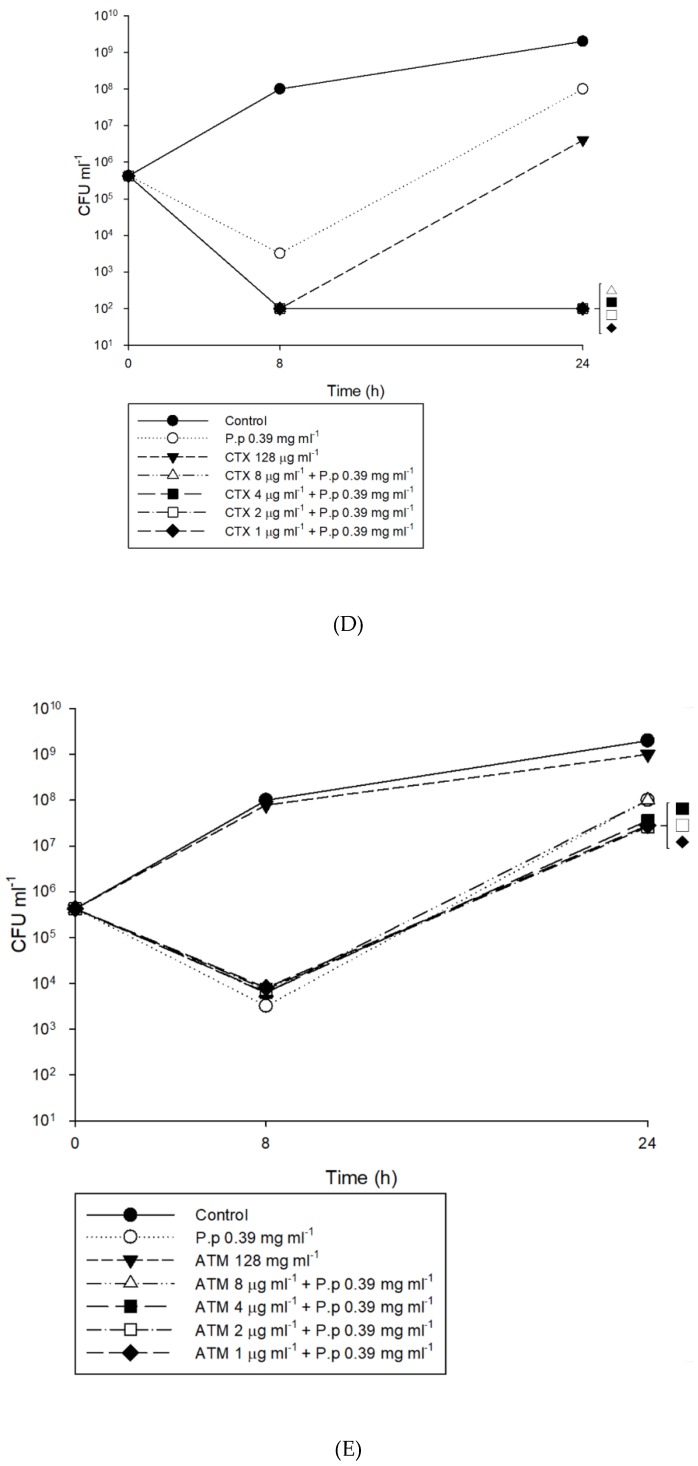

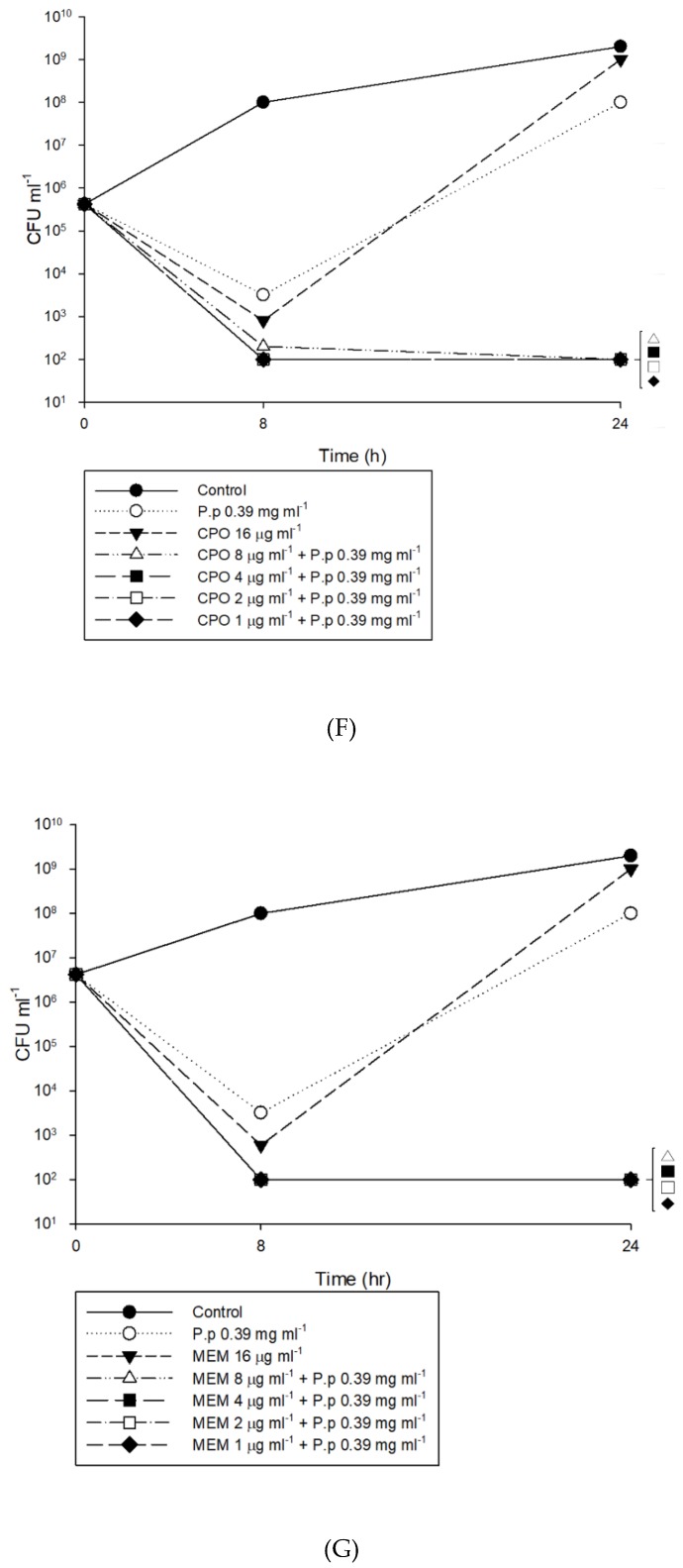
Time-kill results for the crude extract of *Pongamia pinnata* seed coat (concentration: 0.5× minimum inhibitory concentration, MIC) in combination with seven antibiotics including (**A**): ampicillin (AMP), (**B**): cefazolin (CZ), (**C**): cefuroxime (CXM), (**D**): cefotaxime (CTX), (**E**): aztreonam (ATM), (**F**): cefpirome (CPO), and (**G**): meropenem (MEM) against methicillin-resistant *Staphylococcus aureus* 3322 incubated for 48 hr. The concentration used was 1× MIC, and 8, 4, 2, and 1 µg mL^−1^ of each of the different antibiotics were evaluated. The inoculum concentration was 5 × 10^5^ colony forming units (CFU) mL^−1^.

When MRSA 3322 was co-cultivated with 0.5× MIC of *P. pinnata* and 1× MIC (32 µg mL^−1^) of ampicillin, the inhibitory effect persisted for only 8 hours, and then regrowth occurred (Figure 3A). When co-cultivated with 0.5× MIC of *P. pinnata* extract and 1 µg mL^−1^ ampicillin, the inhibitory effect persisted for 8 hours, and then regrowth occurred. When cocultivated with 0.5× MIC of *P. pinnata* extract and 8, 4, and 2 µg mL^−1^ ampicillin, the inhibitory effect persisted for 24 hours. When MRSA 3322 was co-cultivated with 0.5× MIC of *P. pinnata* extract and 1× MIC (64 µg mL^−1^) of cefazolin, the inhibitory effect persisted for only 8 hours, and regrowth occurred (Figure 3B). When co-cultivated with 05× MIC of *P. pinnata* extract and 8, 4, 2, and 1 µg mL^−1^ cefazolin, the inhibitory effect persisted 24 hours before regrowth.

When MRSA 3322 was co-cultivated with 1× MIC of cefuroxime, cefotaxime (128 µg mL^−1^), and cefpirome (16 µg mL^−1^), the inhibitory effect persisted for only 8 hours, and regrowth occurred (Figure 3C,D,F). When co-cultivated with 0.5× MIC of *P. pinnata* extract and 8, 4, 2, and 1 µg mL^−1^ cefuroxime, cefotaxime, and cefpirome, the inhibitory effect persisted 24 hours before regrowth occurred. When MRSA 3322 was co-cultivated with 1× MIC of meropenem (16 µg mL^−1^), the inhibitory effect persisted for only 8 hours, and then regrowth occurred (Figure 3G). When co-cultivated with 0.5× MIC of *P. pinnata* extract and 8, 4, 2, and 1 µg mL^−1^ meropenem, the inhibitory effect persisted 24 hours before regrowth occurred. When MRSA 3322 was co-cultivated with 1× MIC (128 µg mL^−1^) of aztreonam alone or co-cultivated with 0.5× MIC of *P. pinnata* combined with 8, 4, 2, 1 µg mL^−1^ aztreonam, the inhibitory effects of all treatments persisted for only 8 hours before regrowth occurred (Figure 2E). All antibiotics except aztreonam had synergistic effects when combined with *P. pinnata* extract, even at low concentrations.

## 4. Discussion

Herbal medicine is one of the most important fields of traditional medicine in the world [20]. Previous studies have reported on the benefits of *P. pinnata* extracts including the gastro-protective properties of seed extracts in adult male albino rats, the anti-hyperglycemic and anti-lipid peroxidative activities of fruit extracts in diabetic rats, and the antimicrobial activity of bark, seed, and leaf extracts against various bacteria and fungi [21,22,23,24]. In this study, we investigated the in vitro activity of a combination of *P. pinnata* seed coat extract with various antibiotics against MRSA. We found that the crude aqueous extracts of the seed coat of *P. pinnata* resulted in MBC/MIC ratios of < 4. The time-kill assays revealed bactericidal effects at 2× and 4× MIC for the antibiotics tested. These findings indicated that the *P. pinnata* extract has bactericidal effects on MRSA but that high concentrations are required for it to be effective when used alone. On the other hand, when the extract was combined with various antibiotics that were typically not inhibitory to MRSA, we observed synergistic effects between the *P. pinnata* extract and most of the tested antibiotics, even at very low concentrations, except for aztreonam. A previous study found that the combination of vancomycin and β-lactam antibiotics is synergistic against staphylococci with reduced susceptibilities to vancomycin [25]. Most β-lactam antibiotics used alone have no killing effect on MRSA. However, when they are used with an anti-MRSA agent, they enhance its killing effect [26]. Such a mechanism and result may explain our experimental finding of synergy between *P. pinnata* extract and the β-lactam antibiotics tested, which showed enhanced killing of MRSA.

In this study, we did not study the possible active antibacterial component of *P. pinnata* extract. However, previous studies have investigated this issue. Various extracts of the plant exhibited antibacterial activity against a broad spectrum of bacteria. The plant possesses numerous phytoconstituents such as flavones, flavans, chalcone, triterpenes and aromatic carboxylic acids, which are possibly responsible for the antibacterial activity [26,27]. In addition, the toxicity of the extracts of *P. pinnata* has been evaluated in animal studies [28,29]. In acute toxicity studies, rats treated with crude seed extracts of *P. pinnata* at a dose of 2000 mg/kg body weight were safe at 14 observation days [28]. In subacute toxicity studies, the rats were treated orally with *P. pinnata* crude seed extract suspensions at a dose of 1600 mg/kg body weight daily for 20 days, and the test drug did not produce significant toxic effects except mild to moderate pathological changes observed in the spleen and liver [29]. Overall, these findings suggest that *P. pinnata* is relatively safe.

A literature search for studies of *P. pinnata* extracts revealed no other scientific investigations regarding the antimicrobial effects of aqueous seed coat extracts inhibiting antibiotic-resistant bacteria, particularly MRSA. We restricted our study to the aqueous seed coat extract since the antimicrobial activity could be attributed to the synergistic effects of the combination of various components present in the aqueous extract. Further study including chromatography is necessary to clarify the chemical and pharmacological properties of the extract. Based on these results, we suggest that *P. pinnata* has promising antimicrobial activity and synergistic effects with other antibiotics used for the treatment of MRSA. Such plant extracts may be used to develop new pharmaceuticals of natural origin for antimicrobial purposes.

Our study has some limitations. First, the relatively high values of *P. pinnata* MICs in our experiments could be attributed to the crude extracts and unknown active constituents. Second, a greater number of bacterial species need to be tested to confirm the activity of the extract. 

In conclusion, water extracts of *P. pinnata* seed coat have excellent in vitro synergistic inhibitory effects in combination with several antibiotics used against MRSA. The combinations exhibited synergistic in vitro MRSA killing effects. The aqueous extract of the seed coat of *P. pinnata* has potential as an antimicrobial agent for clinical use, especially for the treatment of MRSA. 

## Figures and Tables

**Table 1:** The ratio of minimal bactericidal concentration/minimal inhibitory concentration (MBC/MIC) of *Pongamia pinnata* extract against 10 methicillin-resistant *Staphylococcus aureus* (MRSA) isolates.

Isolates.	MBC (mg mL^−1^)	MIC (mg mL^−1^)	MBC/MIC
MRSA 252	0.78	0.78	1
MRSA 3315	1.56	0.39	4
MRSA 3322	1.56	0.78	2
MRSA 3337	1.56	0.39	4
MRSA 3509	1.56	0.39	4
MRSA 3562	1.56	0.39	4
MRSA 3626	0.78	0.78	1
MRSA 3635	3.12	0.78	2
MRSA 3641	1.56	0.78	2
MRSA 3643	1.56	0.39	4

**Table 2:** The checkerboard microdilution assay of seven antimicrobial agents combined with a crude extract of *Pongamia pinnata* seed coat against 10 methicillin-resistant *Staphylococcus aureus* isolates. The initial concentration of each bacterial suspension was 1.5 × 10^5^ CFU mg mL^−1^. The final drug concentration ranged from 6.25–0.098 mg mL^−1^ for the extract and 256–0.25 µg mL^−1^ for the antibiotics.

Class	Antibiotic	FIC Index			Range	Mean	SD
		> 4	> 0.5, ≤ 4	≤ 0.5			
Penicillins	Ampicillin	0	0	10	0.25–0.5	0.43	0.1
Carbapenems	Meropenem	0	0	10	0.25–0.5	0.36	0.09
Cephalosporins	Cefazolin	0	0	10	0.266–0.375	0.33	0.04
	Cefuroxime	0	3	7	0.266–0.504	0.4	0.1
	Cefotaxime	0	1	9	0.188–0.516	0.33	0.11
	Cefpirome	0	1	9	0.281–0.563	0.46	0.09
Monobactams	Aztreonam	0	10	0	0.75–1	0.98	0.08
